# Patient and healthcare professional perspectives on implementing patient-reported outcome measures in gender-affirming care: a qualitative study

**DOI:** 10.1136/bmjoq-2023-002507

**Published:** 2023-11-08

**Authors:** Rakhshan Kamran, Liam Jackman, Anna Laws, Melissa Stepney, Conrad Harrison, Abhilash Jain, Jeremy Rodrigues

**Affiliations:** 1 Nuffield Department of Orthopaedics, Rheumatology and Musculoskeletal Sciences, University of Oxford, Oxford, UK; 2 Temerty Faculty of Medicine, University of Toronto, Toronto, Ontario, Canada; 3 Northern Region Gender Dysphoria Service, Cumbria, Northumberland, Tyne & Wear NHS Foundation Trust, Newcastle, UK; 4 The CHiMES Collaborative, Department of Psychiatry University of Oxford, Oxford, UK; 5 Centre for Academic Primary Care, University of Bristol, Bristol, UK; 6 Department of Plastic Surgery, Stoke Mandeville Hospital, Buckinghamshire Healthcare NHS Trust, Aylesbury, UK; 7 Warwick Clinical Trials Unit, University of Warwick, Coventry, UK

**Keywords:** Patient-centred care, Implementation science, Qualitative research, Health services research

## Abstract

**Objectives:**

Patient and healthcare professional perspectives are needed to develop a gender-affirming care patient-reported outcome measure (PROM) implementation plan. We aimed to identify top considerations relevant to gender-affirming care PROM implementation from patient and healthcare professional perspectives.

**Design, settings and participants:**

This qualitative study conducted in the UK between January and April 2023 includes focus groups with a patient sample diverse in age and gender identity, and a healthcare professional sample diverse in age and role. Established methods in implementation science and the Consolidated Framework for Implementation Research were used to create interview guides, and analyse data. Focus groups were audio recorded, transcribed verbatim and analysed by two independent researchers. Patient and healthcare professional focus groups were conducted separately.

**Primary outcome measures:**

Patient and healthcare professional perspectives on PROM implementation were explored through focus groups and until data saturation.

**Results:**

A total of 7 virtual focus groups were conducted with 24 participants (14 patients, mean (SD) age, 43 (14.5); 10 healthcare professionals, mean (SD) age, 46 (11.3)). From patient perspectives, key barriers to PROM implementation were mistrust with PROMs, lack of accessibility, burden, and lack of communication on why PROMs are important and how they will help care. From healthcare professional perspectives, key barriers to PROM implementation were lack of accessibility, burden with PROM administration and scoring, costs of implementation (financial and time), and lack of communication on what PROMs are and how they benefit service provision.

**Conclusion:**

Gender-affirming care PROM implementation must address: patient mistrust with PROMs, accessibility, communication on what PROMs are and how they can be used, reducing burden, and hybridised implementation. These factors may also be applicable to other clinical areas interested in implementing PROMs.

**WHAT IS ALREADY KNOWN ON THIS TOPIC**

- Several international calls have been made for evidence-based patient-reported outcome measure (PROM) implementation to improve gender-affirming care. A recent systematic review identifies that there is no literature on the patient perspective to implementing PROMs for gender-affirming care, representing a key barrier to PROM implementation for this area.

**WHAT THIS STUDY ADDS**

- This is the first study to investigate patient and healthcare professional perspectives on gender-affirming care PROM implementation.

**HOW THIS STUDY MIGHT AFFECT RESEARCH, PRACTICE OR POLICY**

- Gender-affirming care PROM implementation must address: patient mistrust with PROMs, PROM accessibility, communication on what PROMs are and how they can be used, reducing PROM burden, and hybridised implementation. These findings can be used by clinicians, commissioners and policy-makers interested in leading PROM implementation initiatives for gender-affirming care with potential generalisability to other clinical areas.

## Introduction

Gender-affirming care includes psychosocial, hormonal and surgical care to help with gender transition.^1^ International standards emphasise that individual patient needs must be comprehensively understood to offer high-quality gender-affirming care.^1^ Patient-reported outcome measures (PROMs) are self-report instruments helping align care with patient needs.^2^ Gender-affirming care could benefit from widespread, systematic and patient-centred PROM implementation. However, research demonstrates PROM implementation for gender-affirming care is inconsistent, does not follow established methods in implementation science and lacks patient centredness.^3^

The existing literature on PROM implementation for gender-affirming care has identified over 200 PROMs used for gender-affirming care.^3^ However, the benefit of these PROMs is limited due to unaddressed implementation challenges.^3^ Rather than develop new PROMs which may contribute to research waste, implementing existing PROMs more effectively to meet current needs is reported to be a more efficient use of healthcare funding and resources.^4 5^ Past literature emphasises the potential for PROMs to improve gender-affirming care quality, if implemented effectively.^6^

Patient and healthcare professional perspectives on implementation barriers and enablers must be understood to create a PROM implementation plan.^7–9^ The Consolidated Framework for Implementation Research (CFIR) is an implementation science ‘meta framework’, combining key implementation science concepts in one framework. The CFIR can guide design and analysis of qualitative implementation studies and comprises of five domains (table 1).^10–12^ The CFIR has been successfully applied to PROM implementation initiatives and includes guidance for developing interview guides, and categorising implementation barriers and enablers.^8 13^

**Table 1:** CFIR domains and definitions from Damschroder *et al*^10^

CFIR domain^10^	Definition
Innovation^10^	The ‘thing’ that is being implemented, for example, PROMs.^10^
Outer setting^10^	The context in which the Inner Setting exists, for example, healthcare system, country.^10^
Inner setting^10^	Where the innovation is being implemented, for example, gender clinics.^10^
Individuals^10^	Roles and characteristics of people, for example, implementation team members, innovation deliverers (ie, healthcare professionals), innovation recipients (ie, patients).^10^
Implementation process^10^	Sequential steps and strategies to implement the innovation.^10^

We aimed to understand patient and healthcare professional perspectives on PROM implementation for gender-affirming care through focus groups. Results can be used to implement PROMs for gender-affirming care.

## Methods

### Reporting

Reporting follows Consolidated criteria for Reporting Qualitative research.^14^

### Patient and public involvement

Six patient and public partners representing members of the transgender and non-binary community, recruited through representatives from national transgender charity organisations and community support groups, were involved in designing and conducting this study. Patient and public partners confirmed the relevance and importance of the research question, were involved with reviewing and pilot-testing focus group interview guides, and confirmed applicability and relevance of findings.

### Research team, reflexivity

Focus groups were conducted by a cisgender male and doctoral candidate at the University of Oxford (RK) with qualitative research training. The researcher is also an MD candidate, with a clinical background. To aid reflexivity, memos and notes were drafted following each focus group to build awareness of positionality and discuss potential challenges and issues that arose from the focus groups. This was a continual process.

### Relationship with participants

A relationship was not established prior to study commencement with participants. Participants knew the researcher identity and research goals. The researcher introduced themselves, reasons for the focus group and data security during focus groups. Participants were provided with contact information if they had additional questions or concerns.

### Methodological orientation

We followed established qualitative implementation science methods from the CFIR.^10 12 15 16^

### Participant selection

Recruitment occurred through an intermediary at the gender clinic (AL) who sent a recruitment email to patients and healthcare professionals on email lists. The email explained the study, time commitment and data security. Patients were purposively selected to maximise diversity in gender identity and age. Healthcare professionals were sampled purposively to maximise diversity in role. Participants received a £40 voucher in line with the National Institute for Health and Care Research guidance for participant reimbursement.

### Setting

Data were collected through virtual focus groups on Microsoft Teams. Only participants and researcher were present.

### Data collection

A focus group interview guide (online supplemental appendix 1) was developed covering gaps from a past systematic review^3^ and key CFIR concepts.^10^ This was pilot tested with a patient and public involvement group (six members from the transgender and non-binary community representing national transgender organisations and support groups) and a healthcare professional (AL). Patient focus groups were conducted separately from healthcare professional focus groups. Focus groups were 1.5 hours and audio recorded. Focus groups continued until data saturation, were not repeated and were transcribed verbatim (RK). Findings were returned to participants for checking, with quotes anonymised with a participant ID, organised according to CFIR domains.

10.1136/bmjoq-2023-002507.supp1 Supplementary data

### Data analysis

Two researchers (RK and LJ) independently analysed transcripts according to the CFIR (examples displayed in table 2) on Microsoft Word (V.16.69) with disagreements resolved through discussion. Data analysis occurred on Microsoft Excel (V.16.69). Rigour was achieved through ongoing deliberation and application of researcher reflexivity, debriefing meetings between researchers (RK and LJ) to cover analysis progress and identifying key concepts from analysis.

**Table 2:** Examples of CFIR categorisation

Text	CFIR domain^10^	CFIR construct^10^	CFIR subconstruct^10^
‘But also some people don't have the money or the access to technology or the Internet, so therefore paper might be a lot easier for PROMs.’—P011	Inner setting^10^	Structural characteristics^10^	Information technology infrastructure^10^
‘I was thinking about whether some people might feel reluctant to engage if they didn't really understand why it [PROM implementation] was being done or…what the purpose of it was…’—S006	Implementation process^10^	Engaging^10^	Innovation recipients^10^

CFIR, Consolidated Framework for Implementation Research.

## Results

A total of 7 focus groups (3 patients, 4 healthcare professionals) with 24 participants (14 patients, 10 healthcare professionals) (table 3) were conducted in January–April 2023.

**Table 3:** Demographic information of focus group sample

Patient characteristics	Frequency (%)
Demographic information	
Age (mean, SD)	43 (14.5)
Gender*	
Male	1 (7%)
Female	9 (64%)
Trans female	1 (7%)
Agender	1 (7%)
Non-binary/genderqueer	1 (7%)
Non-binary	1 (7%)
Sex assigned at birth	
Male	10 (71%)
Female	3 (21%)
Intersex	1 (7%)
Race	
White	13 (93%)
Mixed white/Asian	1 (7%)
Ethnicity	
British	9 (64%)
Scottish	1 (7%)
Mixed British/European/Middle-Eastern	1 (7%)
Mixed British/Irish	2 (14%)
Mixed Russian/Jewish	1 (7%)
Healthcare professional characteristics	
Demographic information	Frequency (%)
Age (mean, SD)	46 (11.3)
Gender	
Female	9 (90%)
Male	1 (10%)
Sex assigned at birth	
Female	9 (90%)
Prefer not to answer	1 (10%)
Race	
White	8 (80%)
Asian	1 (10%)
Mixed white/Asian/black	1 (10%)
Ethnicity	
British	7 (70%)
Scottish	1 (10%)
Chinese	1 (10%)
Mixed	1 (10%)
Healthcare professional role	
Nurse	3 (30%)
Speech and language therapist	1 (10%)
Peer support worker	2 (20%)
Physician	3 (30%)
Assistant psychologist	1 (10%)

*Participants were asked about gender and sex assigned at birth using the two-step method, where participants were first asked their gender and then their sex assigned at birth through an open-ended response

### Patient perspectives on gender-affirming care PROM implementation organised by CFIR domain

#### Innovation

Top considerations to PROM implementation from the patient perspective under the innovation domain were mistrust with PROM administration and scoring, in particular, how PROMs could address wider systemic issues for gender diverse people. In general, patients widely felt unsettled with how PROM scoring may impact care quality and access. Only one participant mentioned not feeling mistrust with PROMs as completing forms is an ‘automatic’ process for them.

I don't have any trust that PROMs are feeding in to change the system or change the approach of everything. It…feels like a paperwork exercise.—P013

A score would unsettle me…. It would also skew my responses if I knew I was being marked… What do I need to say to get treatment? Is this gonna be if I get a 26, ‘Ohh you didn't get 30, you're not getting any treatment because we don't think you qualify.’—P003

I am on the opposite side… I have filled so many…forms out…that it has become an automatic process for me…From what I have heard, this is a very unique perspective.—P006

Participants were concerned about lack of PROM accessibility. Specifically, PROMs being inaccessible to people with neurodivergence, and the need for large print, simplified language, multiple languages and high contrast versions of PROMs. PROM burden (PROM length, time needed to complete and repetitive questions) concerned some participants.

Accessibility is always the biggest thing. So, if English isn't the first language, dyslexia, if they've got difficulties reading…, if they've got sight issues.—P011

I did notice…an awful lot of repetition… I think I would find it difficult not to put a line through it (PROM) and throw it away.—P005

#### Outer setting

A widespread perspective on enabling PROM implementation under the outer setting domain was positioning PROMs as a way to hold the National Health Service (NHS) accountable for providing high-quality care. Patients mentioned increased motivation to complete PROMs if they would improve their care.

The idea that the clinician, the clinic, the NHS is being held…accountable through the PROMs…would make people want to fill them in…—P012

Some patients were concerned about who PROM data is shared with. Specifically, patients with negative general practitioner (GP) interactions worried PROM completion would negatively impact care. Patients with positive GP interactions were also concerned that sharing PROM data with GPs would limit interim care received by their GP.

I would definitely not fill a PROM in before or after a clinic meeting. Not a hope. To know this would go back to my wait time, primary GP surgery horrifies me, after the damage they have done to me.—P004

It’s difficult to access interim care through a GP whilst you're waiting for…support from a gender clinic. It’s…seen as they've handed the job on, and I think if the information is being shared directly with some GP’s… it might be seen that you're already engaging with the process. Therefore, they don't need to do anything…So I think the audience for the information is really important that we [patients] get a choice about…—P005

Patients also reported mistrust with PROMs were related to the negative political environment around gender-affirming care. Patients felt it was important PROM implementation did not add to waiting times. Some participants reported completing PROMs was a dehumanising experience.

What it comes down to…is [completing PROMs] dehumanizes the person that you're asking to fill in the form.—P009

#### Inner setting

The most widely encountered consideration under the inner setting domain was lack of communication on information about PROMs. Key questions patients wanted answered were: why PROMs are being administered, how PROM responses impact care, and how PROMs benefit patients. Lack of communication on PROMs contributed to mistrust with PROMs. Hybridised PROM implementation (ability to complete online or in-person) was also supported.

And there’s…no information… Whenever I've got PROMs, it’s…like this is a form - fill it out and give it back to us now…that’s it.—P013

People are more likely to want to help their own care. I think it’s…an explanation at the top, which…it’s a lot of information…but…necessary. Either having a paragraph or a QR code to a video and explaining this is what a PROM is, this is why we're collecting…information, this is the confidentiality, this is the data breach… And I think there should definitely be a mix of both [online and in-person administration] because some people wouldn't want to do it sat in the clinic with the time pressure. But also, some people don't have the money or…access to…the Internet…—P011

Patients felt PROM implementation should be tailored to the needs of patients. For example, incorporating patient preferences on how they would like to be communicated with. Patients also widely reported PROM implementation would be enabled with adequate space and time to complete the PROM.

It is a difficult one with PROMs because they are going for personal questions. It needs to be an environment where you can ask for help…but if you want that privacy, the helper leaves the room…—P003

#### Individuals

Under the individuals’ domain, patients felt having peer support staff at the gender clinic available if PROM completion was distressing was an important safeguard. This concept was important to patients as it was a widespread belief that PROMs asked sensitive questions. A strategy to enhance PROM accessibility was having clinics partner with local organisations. One participant mentioned they use an organisation to help them with completing forms. Other participants agreed that partnering with local organisations may enable PROM implementation.

Just letting them [patients] know that if any gender service has a peer support network…that’s available if anything on the PROM is more distressing to them…—P011

I'm autistic and have ADHD, and I personally sometimes struggle to fill in forms. Pointing people to some organizations that could be of help might be useful. So, Citizens Advice is the most neutral one, but there could also be some like LGBT specific ones…—P014

#### Implementation process

A widespread consideration held by patients under the implementation process domain was assessing how often they would like to complete PROMs. Patients emphasised PROM implementation should reinforce that patients matter over the PROM itself. One way to communicate this is through thinking about the person behind the PROM and assessing their needs.

You have to…take the PROM and say…I'm not asking the computer to fill this in - I'm asking a person to fill this in. So ‘what does that individual person need?’ Not ‘what does the PROM need?’ Because the PROM shouldn't be the thing that we're worried about, it should be the person that’s filling it in.—P009

In addition, some patients mentioned the importance of PROM administration timing. Specifically, some patients mentioned lower motivation to complete PROMs immediately following a distressing appointment. Some patients also mentioned that PROM implementation could be enabled if clinicians helped to explain the PROM as part of the implementation process.

Immediately after…you just had your appointment, ‘Here’s a PROM’ wouldn't work because for quite a lot of people, the sessions that they go to are quite distressing and emotional, and that’s not something you want to immediately put yourself into doing is filling in a PROM.—P011

Online supplemental appendix 2 provides additional quotes organised by three major themes; online supplemental appendix 4 provides additional quotes organised by all CFIR constructs represented.

### Healthcare professional perspectives on gender-affirming care PROM implementation organised by CFIR domain

#### Innovation

In general, healthcare professionals reported PROM complexity was a key barrier to implementing PROMs. Participants were concerned about PROM length, uncertainty about when and how often to administer PROMs, and PROM administration and scoring burden. Automation of scoring with graphical display of results was widely mentioned as an implementation enabler. In additional healthcare professionals felt adapting PROMs to patient accessibility needs was important.

It does add to the complexity and the burden of the consultation for the patient and for us [clinicians] as well because it’s another thing to talk about and it’s already quite a complicated consultation to start with…And I think that’s OK, if there’s some really clear usefulness of it…Also if scoring is done as something that we could click on and see the whole of the graph and how it’s working out, that'd be fantastic. If it was another thing that we had to hunt through billions of documents to find and understand the process before we started the work, that would just be a burden.—S010

Making PROMs accessible to all groups, including people with intellectual disabilities or lower literacy skills, or making easy read versions is important.—S005

Healthcare professionals were also concerned about implementation costs. A few participants were concerned about the cost to the clinic’s reputation if implementation was unsuccessful.

It’s…these things that are unseen and people don't…think about the doctor’s time, the clinical time it costs for the person to sit and explain it to them, the cost…to send out any surveys. And the cost of the paper, the cost of the letter, the cost of postage returned, the time too, and if it is going to be taken from one system to another, if it has to be done manually, then that’s another person’s time.—S002

#### Outer setting

Under the outer setting domain, healthcare professionals generally felt that the political environment of gender-affirming care may pose barriers to PROM implementation. Specifically, there were widespread beliefs that engaging patients to complete PROMs might be difficult due to feelings of mistrust with clinicians stemming from the current political environment.

There is always paranoia with what you are going to do with this really personal information of mine. I see that’s increased over recent times and I think it’s because of the stuff that happens within politics and the media at the moment. People…are much more on hyper alert for that.—S005

Some healthcare professionals felt a barrier to PROM implementation was uncertainty of how to handle responses if patients scored high in PROM sections. For example, if a patient was sent a PROM remotely and scored high on a scale measuring psychological distress. Some participants mentioned the benefits of having an open text box to capture patient comments at the end of a PROM. However, other healthcare professionals felt this would contribute to the uncertainty of how to handle critical PROM responses.

If a PROM is sent out beforehand, somebody completes it, sends it back to admin and then it looks very challenging if lots of things are scored highly on - then it has to be from a risk perspective, and a duty of care, would then be having to end up dealing with it before the patients actually arrive for the consultation.—S008

The problem with [open text box for comments at the end of PROM] is if the patient writes, ‘I'm going to kill myself’. You know what? We're gonna pick that up and what are we gonna do with that?—S010

#### Inner setting

Under the inner setting, it was important for healthcare professionals to have communication on what PROMs are, how they can be used to guide clinical care and the benefits PROM implementation brings to care provision. Participants mentioned failing to communicate these key concepts pose barriers to PROM implementation. It was also widely believed that hybridised PROM implementation would be important for implementation success.

Making it clear how PROMs are going to benefit and help and why we're collecting this data and making it clear like we're not just collecting it for fun, what we're trying to achieve from it, I think will really help.—S004

Having electronic and hard copy available is important, because not everyone has an e-mail or wants to use the computer or can afford Internet.—S007

A few healthcare professionals mentioned issues around PROM data security. Specifically, PROMs being sent to unintended recipients. PROMs taking away clinic time was also an important consideration to PROM implementation mentioned by most participants.

You have to be really careful not to out a patient. So, if you sent it to an old address and they and they opened it, then they might say, ‘Oh my goodness, I didn't realise they went to a gender clinic’.—S010

It is looking at appropriate use of time and it’s [PROMs] going to take time away from…face-to-face contact with clients.—S004

#### Individuals

Under the individuals’ domain, healthcare professionals mentioned the need for staff to facilitate PROM implementation. Some participants mentioned that assistant psychologists and administrative staff could form part of the PROM implementation team. An assistant psychologist felt PROM implementation could form a part of their responsibilities.

There does need to be human behind PROMs, so it doesn't feel like we’re just cold robots asking for your data. This [PROM implementation] kind of aligns with the assistant psychologist job.—S004

It was also mentioned by some participants that senior management buy-in may facilitate implementation.

You need to get to senior management buy into this. So, it’s not just seen as something that our that little Gender Clinic’s gone off on a tangent again and done something a bit different.—S002

#### Implementation process

Under the implementation process domain, participants emphasised the importance of patient engagement. It was mentioned by some that communicating with patients benefits of PROMs and why they are being implemented could facilitate higher engagement. Some healthcare professionals mentioned a strategy to increase patient engagement is confirming patient accessibility needs and ensuring PROMs were accessible.

Some people might feel reluctant to engage if they didn't really understand why it [PROM implementation] was being done or…what the purpose of it was…there would need to be some explanation…for people.—S006

It would be worth having…a question say, ‘Do you have any specific needs? Do you need the PROM adapted into specific formats? Please let us know what you would like and then we can change the text, send different text size, different colour.’…so asking the person first.—S003

Some healthcare professionals felt PROM implementation is a continuous, iterative process and emphasised importance of regular feedback from patients on PROM implementation.

It would be good to have a regular focus group with patients, like, every six months…just to see what they think. Because with something like this, it’s not ever gonna be just one solution or one like a one-time thing. It needs to be sort of a continuous evolution.—S004

Online supplemental appendix 3 provides additional quotes organised by three major themes; online supplemental appendix 5 provides additional quotes organised by all CFIR constructs represented.

## Discussion

This study identifies considerations relevant to PROM implementation for gender-affirming care from patient and healthcare professional perspectives. A recent systematic review identified a lack of literature on patient and healthcare perspectives on PROM implementation and our study fills this gap.^3^

Patient and healthcare professional perspectives on PROM implementation demonstrated overlap. Both groups emphasised addressing the following for PROM implementation: PROM accessibility (accessible to people with neurodivergence; multiple languages, large print and high contrast versions); communication on what PROMs are, their importance and how they can be used to improve care; hybridising implementation; and reducing burden. These key considerations may not be gender-affirming care specific and could also apply to other clinical areas interested in implementing PROMs.

Our results are in line with past literature reporting on healthcare professional knowledge about PROMs as important for PROM implementation,^17^ PROM implementation being a continuous process^18^ and reducing PROM burden to facilitate implementation.^19^ Using computerised adaptive testing has reduced PROM the implementation burden in other clinical areas.^20 21^ However, no PROM implementation studies currently exist in for gender-affirming care and our study fills this gap. The findings from our study can be used to help guide implementation of PROMs for gender-affirming care. Over 200 PROMs have been identified for gender-affirming care^3^ and the findings from this study can help to maximise their uptake, helping to ensure the optimal potential for PROMs are reached, and effecient use of healthcare funding and resources.^4 5^ The results from our study can also help to maximise the potential benefit of PROMs for gender-affirming care.^6^

Several considerations specific to gender-affirming care PROM implementation were covered in this study. First, communicating with patients and healthcare professionals about why PROMs are being administered and how scoring works prior to PROM administration. This was related to a key theme regarding trust with this population. Second, confirming patient accessibility needs prior to PROM administration. Partnering with local and LGBTQ+ organisations was mentioned as strategies to increase PROM accessibility. Third, it is important to confirm with patients who they consent to have their PROM data and results shared with. A practical consideration to reduce the risk of PROMs sent to unintended recipients is implementing multifactor authentication for remote PROM completion—this would be important given the theme of trust and fears regarding data privacy. This consideration has been used in other settings with remote patient monitoring.^22^

Strengths of this study include: a patient sample diverse in age and gender identity, a healthcare professional sample diverse across interdisciplinary roles and application of established methods in implementation science.^10 15^ Using CFIR to structure the study lends to developing real-world implementable strategy solutions.

Limitations include lack of racial and ethnic diversity in the sample. Future research should aim to seek perspectives from groups not represented in this study (ethnic minority trans patients and those experiencing multiple marginalisation’s within healthcare). Survey studies using open-ended responses may provide methods to capture perspectives in larger samples of people.^23^

This study provides practical recommendations for PROM implementation for gender-affirming care. These include: improved communication on PROMs and rationale for implementation, ensuring PROMs are accessible to patient needs, and ensuring PROM results are only shared with individuals patients consent to have PROM results sent to. Further, hybridising PROM implementation and identifying staff who can help facilitate implementation (ie, administrative, assistant psychologists) may maximise PROM implementation. Further studies may seek to qualitatively explore the most acceptable PROM to use for different gender-affirming care clinical settings.^3 24^

## Conclusion

PROM implementation for gender-affirming care must be patientcentred and address key concepts important to healthcare professionals for successful and sustained PROM implementation. The main considerations for PROM implementation include: patient mistrust with PROMs, PROM accessibility, communication on what PROMs are and how they can be used, reducing PROM burden, and hybridised implementation. These considerations can be used to help guide implementation of one of the over 200 PROMs identified for gender-affirming care, ensuring efficient use of healthcare resources and improved quality of gender-affirming care delivery.

## Data Availability

All data relevant to the study are included in the article or uploaded as online supplemental information. RK had full access to all the data in the study and takes responsibility for the integrity of the data and the accuracy of the data analysis.
