# Supplementary material for: Patient and healthcare professional perspectives on implementing patient-reported outcome measures in gender-affirming care: a qualitative study
Source: BMJ Open Qual. 2023 Nov 8;12(4):e002507. doi: 10.1136/bmjoq-2023-002507 (PMC10632877; doi:10.1136/bmjoq-2023-002507)
Supplement: Supplementary data [file bmjoq-2023-002507supp001.pdf]

BMJ Publishing Group Limited (BMJ) disclaims all liability and responsibility arising from any reliance placed on this supplemental material which has been supplied by the author(s)

Supplementary Material Table of Contents

Appendix 1……Focus Group Interview Guide

Appendix 2…………………………………………………………………………………………...…….3 Major Themes and Supportive Quotes from Patient Focus Groups

Appendix 3………………………………………………………………………………3 Major Themes and Supportive Quotes from Healthcare Professional Focus Groups

Appendix 4…………………………………………………………………………..…Additional Patient Supportive Quotes on Gender-Affirming Care PROM Implementation

Appendix 5…………………………………………………………..Additional Healthcare Professional Supportive Quotes on Gender-Affirming Care PROM Implementation

Appendix 1. Focus Group Interview Guide

Patient Focus Group Questions*

1. What do you think about using a PROM in clinic?
2. Thinking about the PROM, would you want to complete something like this at your appointment at a gender clinic?
    a) Why/why not?
    b) If not, do you have suggested changes to the form that would change your answer?

Barriers and enablers to PROM completion

3. You might be asked to complete a PROM before coming to clinic, in clinic (e.g., in the waiting area), or after a clinic appointment while on the waiting list for treatment. Taking each of these in turn, what do you think would make it easier for you to complete the form? For example, having your clinician ask you to explain the PROM, having your clinician explain how results will be used, information on how your form responses will be confidential…
    a) Probe for reasons why: i.e., feeling safe to complete the PROM
    b) Is this a view shared by others here? Do you feel the same or differently about this?
4. Thinking again of the situation where you might be asked to complete a PROM like this for your gender clinic appointment, what what might stop you completing the PROM? For example, feeling unsafe to complete the PROM, not having a private location to complete the PROM, feeling like the PROM will be a waste of time…
    a) Probe for reasons why: i.e., accessibility, people feeling like their results won’t be used

b) Is this a view shared by others here? Do you feel the same or differently about this?

Suggested adaptations to the PROM

5. Is there anything we can change about PROMs so that so that it is easier for you to complete? For example, a message about how your PROM information will be kept secure, or ways to administer the PROM online?

6. Is there anything we can change which would encourage or help you to answer all of the questions in a PROM?

Suggestions on PROM implementation – logistics of completion

7. When would you like to complete the PROM (for example: before and after every gender clinic appointment, only for some appointments, etc.)?

    a) Prompt for: burden on frequency of completion, when patients view it is most critical to measure outcomes, certain times when it may not be a good idea to complete the PROM

8. Where would you like to complete the PROM (i.e., at home, or in clinic or other location)?

    a) Prompt for: reasons why participants have a preference for a certain location, if they would need any supports to complete the PROM at these locations, why it would be easier/more difficult in certain locations

Suggestions on PROM implementation – burden and form administration

9. How long would you be willing to spend to complete the PROM (i.e., 5-10 minutes, 10-20 minutes)?

10. How would you like to complete the PROM (i.e., pen and paper, iPad, on own device, a device from clinic)?

Thoughts on score communication

11. After completing the PROM, the results can be used to show a score. Would you be interested in seeing your scores? If we sent you the scores, how would like us to do this?

Thoughts on supports needed

12. If you could have support filling out this PROM, what would you like (i.e., online resources, specific instructions, contact information for mental health support)?

Final thoughts/comments

13. Do you have any final thoughts or comments to add?

*PROM used as an example for focus group discussions was the Gender Congruence and Life Satisfaction Scale (GCLS), used with permission from developers and available at: Jones BA, Bouman WP, Haycraft E, Arcelus J. The Gender Congruence and Life Satisfaction Scale (GCLS): Development and validation of a scale to measure outcomes from transgender health services. Int J Transgend. 2018 Apr 26;20(1):63-80. doi: 10.1080/15532739.2018.1453425. PMID: 32999594; PMCID: PMC6831013.

Healthcare Professional Focus Group Questions*

Barriers and enablers to PROM implementation

1. What are your initial thoughts about implementing PROMs at your clinic?

2. One way that clinics implement PROMs is through setting up an electronic system. The electronic system can contact patients once they are referred to a clinic but before they come in. Patients can then complete the PROM and have their responses scored. What are your thoughts on having a system like this for your clinic?

    a) Prompt for: what barriers might you encounter implementing this in your clinic?

    b) Would limited resources or culture issues make implementing this system a challenge?

3. If your team wanted to use PROMs regularly with patients in clinic, what are the barriers or likely issues you anticipate? For example: disruption to clinic flow, burden on clinicians to administer and score the PROM, lack of information on how to use PROM responses to improve care.

4. Sometimes it can help motivate patients to complete PROMs. For example, if clinicians encourage patients to complete the PROM or patients see their results being used to guide or improve care.

    a) What might be the barriers to encourage patients to complete PROMs in your clinic?

    b) What would help to make this easier to do?

5. Another helpful strategy can be having patients see how their PROM results are being used to guide or improve care.

a) What might be the barriers to showing patients how their PROM results are being used for their care?

b) What would help to make this easier to do?

6. What would make it easier for you and your clinic to implement PROMs for day-day use?

Suggestions on PROM Implementation – logistics of completion

7. PROMs can be administered electronically to patients, or by pen-and-paper, for example by having a receptionist hand the PROM to patients when they arrive, and collecting the PROM before going into their appointment. Can you describe how you think PROMs should be implemented into your clinic?

a) How will it interact or conflict with current programs or processes?

8. When do you think PROMs should be completed (for example: before and after every gender clinic appointment, only some appointments, etc.)?

a) Probe for reasons why: clinician or patient burden in administering the PROM, data handling (i.e., more data to integrate with increased frequency of completion), the most important clinical timepoints which would be useful to measure PROMs?

Thoughts on how PROMs will be used

9. How do you envisage your clinic using PROMs data?

a) Probe: using PROM responses to improve care?

10. How will your clinic keep PROM responses confidential so that patients do not worry about critical responses having a potential impact on their care?

Ability to integrate PROM into current workflow

11. How might PROMs fit with your existing work processes and practices in your setting?

12. What kinds of changes or alterations do you think need to be made to PROMs so it will work effectively in your setting?

    a) Do you think you will be able to make these changes? Why or why not?

Costs of implementation

13. What costs do you think should be considered when deciding to implement the PROM?

Impact of clinic infrastructure and culture on PROM implementation

14. How do you think the infrastructure of your clinic (composition of staff, age, maturity, size, or physical layout) facilitate or hinder the implementation of PROMs?

    a) What are ways you think these structural challenges can be overcome?

    b) What kind of approvals will be needed? Who will need to be involved?

c) Can you describe the process that will be needed to make these changes?

15. How do you think your clinic's culture (general beliefs, values, assumptions that people embrace) will affect the implementation of PROMs?

Resources for PROM implementation

16. Do you expect to have sufficient resources to implement and administer PROMs?

a) What resources are you counting on? Are there any other resources that you received, or would have liked to receive? What resources will be easy to procure?

Self-efficacy for PROM implementation

17. How confident are you that you will be able to successfully implement PROMs? What gives you that level of confidence (or lack of confidence)?

Final thoughts/comments

18. Do you have any final thoughts or comments to add?

*PROM used as an example for focus group discussions was the Gender Congruence and Life Satisfaction Scale (GCLS), used with permission from developers and available at: Jones BA, Bouman WP, Haycraft E, Arcelus J. The Gender Congruence and Life Satisfaction Scale (GCLS): Development and validation of a scale to measure outcomes from transgender health services. Int J Transgend. 2018 Apr 26;20(1):63-80. doi: 10.1080/15532739.2018.1453425. PMID: 32999594; PMCID: PMC6831013.

Appendix 2. 3 Major Themes and Supportive Quotes from Patient Focus Groups

Major Theme	Supporting Quotes
**Innovation (PROM): Source, Design, Accessibility, Complexity**	Of the amount of trust we're placing in the information we give and how it's to be used, it's so important to moving forward because I feel a lot of people will just walk away from it. Just say, 'No thank you' and put a line through it. And it's rightly said. This is what's got to be addressed. You've got to have trust with PROMs. And as I say, and I absolutely agree, trust permeates to us. But unless you can build that trust up, and it really does need building up, then it's just not going to be there. - P004
	I think scoring would skew things heavily towards exaggeration, lying and manipulation of the PROM. If you're giving me a score, what's that for? And if it's not for assessment, why are you giving me this? Because now I feel like it's an assessment. And it does become very - I'm playing a game now. I'm not answering the questions. But that's how it feels to me, it's I need to win. Because I know I need to get this form filled in, so you give me the 30 or whatever the threshold is. And that's how I would see it if you said that I was getting a score at the end. - P011
	I think that there's a lot of work showing differences in the way neurodivergent people respond to questionnaires and also specific difficulties with ambiguous wording that most neurotypicals resolve to the same meaning and neurodivergent people tend to sit and worry which meaning they should respond based on that. So, I guess that really that's really about the accessibility of the questions themselves. Have they been written in a way that takes account of those kinds of differences? – P012
	One of the things that I did notice is an awful lot of repetition. And I know that you ask a question in several different ways to try and get to the root of an answer, but I did notice that I think I would find it difficult not to put a line through it and throw it away. - P005
**Outer Setting: Local Attitudes and Inner Setting: Communication**	Definitely trust and transparency from the NHS and wider government is at an all-time low for trans people. - P003
	I just want to recall that it's become obvious that there is a very big need for transparency, especially around why PROMs are needed. - P006
**Individuals: Implementation Team Members and Implementation Process: Assessing Needs**	Just letting them [patients] know that if any kind of gender service has a peer support network, letting them know that that's available if anything on the PROM is more distressing to them, there's people that they can talk to. - P011
	It is about person-centred care. I mean, I understand the need to have robust clinical tools that can be applied across and that can actually show differences between

	people pre- and post-interventions, but it's about person-centred care, it's always gonna boil down to that. Essentially that people will have different needs, different accessibility needs that kind of thing and they will need to be asked what they are. - P008

Appendix 3. 3 Major Themes and Supportive Quotes from Healthcare Professional Focus Groups

Major Theme	Supporting Quotes
**Innovation: Scoring and Complexity and Outer Setting: Critical Responses**	I will say that a PROM needs to be easy to add up and calculate. I've done some of the psychological measures before, where, like, oh, my God, you need a PhD in maths to try and figure out what the score is. I would like a PROM to be very straightforward and easy to score. I also used one and you had to go online and score it. It was honestly a nightmare. – S008
	Making PROMs accessible to all groups, including people with intellectual disabilities or lower literacy skills, or making easy read versions is important. – S005
	The problem with [open text box for comments at the end of PROM] is if the patient writes, 'I'm going to kill myself'. You know what? We're gonna pick that up and what are we gonna do with that? – S010
**Inner Setting: Communications, Available Resources**	I think basically every single possible way that you can communicate on what PROMs are and what they are used for, you need to communicate it because people learn and take in information in very different ways and so I think you need to reinforce that message in every possible way that you can at every opportunity that you get. So, if you're sending out written information, it needs to be a paragraph. If you need more information, click on this link and you can watch a video. If you'd like to talk to someone about this, then please call. If you have any face-to-face meetings then it needs to be the first thing that you say is just to reiterate, make sure that everybody is aware that this is what this is, what it's for. This is why we're doing it, and if you have any issues with that then please you know. Communicate with us about it and let us help you. So every possible way that you can.-S002
	Unfortunately, I think a lot of the time, the system doesn't support it, and it's not 5 minutes, it's 10 minutes. It's 15 minutes. And then if the system is down for any length of time, that means going back after your next appointment trying to remember what was said at the first appointment, so you review the PROM and that means your day is extended by half an hour, an hour, whatever. If you don't do it that same day, then it gets taken over to the next day. If you only work part time, it gets taken over to the next week and then suddenly you get this snowball where you know I can see that that would be a concern. – S002
**Individuals: Implementation Facilitators and Implementation Process: Engaging**	Or I don't know if admin could support with this. I think if we are going to send out paper copies, there will need to be admin support to send those out at the appropriate points and depending on how often we decide for these to be done-S003

	Having patients feel as though they're part of it. They'll benefit from it. And if they are gonna gain from this, then they will feel more engaged to do it and take part in the PROM, where the thing it's just us doing it for the sake of it, for the number crunch and it will be very hard for them. – S003

Appendix 3. Additional Patient Supportive Quotes on Gender-Affirming Care PROM Implementation

CFIR Domain	CFIR Construct	Sub-Construct	Supporting Quote(s)
**Innovation**	**Innovation Source**	**PROM**	I don't have any trust that PROMs are feeding in to change the system or change the approach of everything. It just feels like a paperwork exercise. Don't trust that it's going to feed in and improve the system for trans people at all. – P013
			I think it is [PROM implementation] kind of a step back. Personally, I am quite suspicious of the consumers of this kind of information, on what the motives are for using them. - P005
			Of the amount of trust we're placing in the information we give and how it's to be used, it's so important to moving forward because I feel a lot of people will just walk away from it. Just say, 'No thank you' and put a line through it. And it's rightly said. This is what's got to be addressed. You've got to have trust with PROMs. And as I say, and I absolutely agree, trust permeates to us. But unless you can build that trust up, and it really does need building up, then it's just not going to be there. - P004
		**Score**	Yes, a score would unsettle me a little bit. It would also skew my responses if I knew I was being marked at the end of it. I would be seeing this more as, 'What do I need to say to get treatment?' Is this gonna be if I get a 26, 'Ohh you didn't get 30, you're not getting any treatment because we don't think you qualify'. - P003
			I think scoring would skew things heavily towards exaggeration, lying and manipulation of the PROM. If you're giving me a score, what's that for? And if it's not for assessment, why are you giving me this? Because now I feel like it's an assessment. And it does become very - I'm playing a game now. I'm not answering the questions. But that's how it feels to me, it's I need to win. Because I know I need to get this form filled in, so you give me the 30 or whatever the threshold is. And that's how I would see it if you said that I was getting a score at the end. - P011
			I totally understand what was said regarding the score. Having been put back many times and refused to be allowed to move on for GRS SRS surgery. You get to a point where you think what the hell have I got to do? - P004
			Yes, I was just very surprised when you said there was a score. I had absolutely no idea. What do you mean by a score? Is it a number? Literally, that's gonna come out of this like 42 in the Hitchhiker's Guide to the Galaxy or something what? - P001
			Understanding the consequence of the scores is important, it kind of implies that there's a pass or a fail, and I think one of the things that builds up over time often is when people get experiences of answering things in a certain way, they will pass on anecdotal information to other people about how you should answer the questions in order to get what you want. - P005

			I talked before about sort of answering strategically, but also you know again this trust in the system like I'm worried that if you do complain, it's like oh, look at this, we've got an awkward patient, so they'll go to the back of the list. So you sort of wanna answer and please them [clinician]. It's all about feeling that you've got to answer in a certain way. You've got to say something to make sure that you're not seen as an awkward patient or not really trans or whatever - P013
			I am on the opposite side… I have filled so many…forms out…that it has become an automatic process for me…From what I have heard, this is a very unique perspective. – P006
	**Innovation Design**	**Accessibility**	Accessibility is always the biggest thing. So if English isn't the first language, dyslexia, if they've got difficulties reading in general, if they've got sight issues. - P011
			And online, it's a lot easier accessibility-wise because you can change brightness, you can change sizes on screens and you can get everything to be read out to you, if that is easier. - P008
			I think that there's a lot of work showing differences in the way neurodivergent people respond to questionnaires and also specific difficulties with ambiguous wording that most neurotypicals resolve to the same meaning and neurodivergent people tend to sit and worry which meaning they should respond based on that. So, I guess that really that's really about the accessibility of the questions themselves. Have they been written in a way that takes account of those kinds of differences? – P012
	**Innovation Complexity**	**PROM**	One of the things that I did notice is an awful lot of repetition. And I know that you ask a question in several different ways to try and get to the root of an answer, but I did notice that I think I would find it difficult not to put a line through it and throw it away. - P005
**Outer Setting**	**Local Conditions**	**Political Environment**	Definitely trust and transparency from the NHS and wider government is at an all-time low for trans people. - P003
			Not only do we have to live with our gender dysphoria, but we are living in a time with an increasingly hysterical anti-trans moral panic. - P002
			Trans health care is just in a huge crisis at the moment. The waiting list, I would say the biggest part of it like getting through one of those places, is just impossible. And if you're not getting through them, then they are not getting healthcare. So I really feel like accountability should start like from the moment someone's referred, like they should be accountable from that moment, and it shouldn't just like, shrug and dismiss people. - P014
			Well, I think that a lot of the really big problems that gender clinics have faced, certainly from the point of view of like, you know, delivering good care, has been a failure to listen to patients and a failure to establish trusting relationships with patients. - P012

	**Local Attitudes**	**GP**	My experience of my GP's, and other people I've spoken to are in a similar position, is that it's difficult to access interim care through a GP whilst you're waiting for direct support from a gender identity clinic. It's kind of seen as they've handed the job on and I think if the information is being shared directly with some GP's, I'm not generalising about everyone, but I know this is fairly common, that it might be seen that you're already engaging with the process. Therefore, they don't need to do anything. It's more evidence that you know well, 'Why are you asking me for stuff, you're already being dealt with by the [gender service]' or whichever Gender Identity Clinic it is. So I think the audience for the information is really important that we get a choice about. Who it's shared with, and what it's being used for? It's just to understand really where we're coming from I guess. - P005
			I would definitely not fill a PROM in before or after a clinic meeting. Not a hope. To know this would go back to my wait time, primary GP surgery horrifies me, after the damage they have done to me. - P004
		**Experience**	What it comes down to, is an issue with all forms, particularly with the NHS is that it dehumanises the person that you're asking to fill in the form in. – P009
	**External Pressure**	**Performance Measurement Pressure**	I feel like the idea that the clinician, the clinic, the NHS is being held in some way accountable through the PROMs would be the thing that would make people want to fill them in more. - P012
			If there was an obligation on the clinicians to abide by the results of the PROM, I'm not sure how exactly it could be implemented, but like if this goes lower than this score, then we should like aim to bring them up by this date, for example. - P014
**Inner Setting**	**Communications**	**General**	And then also, we did discuss about sending outcome measures to people on the waiting list, and it's partially good because you get in all that information while they're not being seen, but also a lot of people find it quite distressing - getting emails or letters from the Gender Identity Clinic thinking there's an appointment and that they've been referred, or something's changed with the hormones and everything, only for it to be just a questionnaire, which isn't something that I thought of until that was brought up. So that was a really interesting point to consider. - P011
			And there's just no information at all. Whenever I've got PROMs, it's just like this is a form - fill it out and give it back to us now. And that's it. - P013
			But you asked would I want to get this before my first appointment. I have been waiting for so long with little to no communication, if I just got this form with little to no context, obviously I know about PROMs because of this focus group, but if I got this PROM without context, I'd be like, they are marking me to put me in place. I want to speak to someone, I do not want to fill out this survey. So if I got this before, I would actually be irritated, and out of context of me not knowing about this, if I were

			to get this in the post I would be saying they are measuring us, why is this the case? - P003
			I just want to recall that it's become obvious that there is a very big need for transparency, especially around why PROMs are needed. - P006
		**PROM Information**	It might be worth putting on the PROM like, 'this is to understand this, and it will be used this way' - P003
			It is not clear what the objective is of the PROM. Whether we are trying to understand the condition, assess the service, or assess the patient and their possible benefits from the service. I think if you could make that clearer in the initial paragraph, I think a patient would be more comfortable in understanding what was required in their responses. - P001
			It's political. It's like we wanna know that if we're filling out stuff that's to do with healthcare, who is using this, what is it being used for, you know - to help improve patient care or is it being used to drive policy in a political way, to try and save money, to try and cut corners? – P013
			People are more likely to want to help their own care. So yeah, I think it's just an explanation at the top, which obviously it's like it's a lot of information to give, but it's necessary. So yeah, either having a paragraph or like a QR code to a video and just kind of yeah, explaining this is what a PROM is, this is why we're collecting the information and then kind of like you explained at the start - this is the confidentiality, this is the data breach. - P011
			I think what would be really good is if the PROMs had, like a tangible end goal. For example, if they say this will be used to produce a report that will be used for this, I think. The more factual it is, the better. – P014
		**Tailoring**	Do you wanna be contacted by e-mail? By post? By text message? Would you like a face-to-face appointment? That kind of thing to discuss your results if that's something that somebody would be interested in, and again if there's time available. - P011
			Reminder messages and say if you've still got this PROM, we'd like it back by this point and reminders might help people. - P005
	**Structural Characteristics**	**Physical Infrastructure and Information Technology Infrastructure**	I think there should definitely be a big mix of both [online and in-person administration] because some people wouldn't want to do it sat in the clinic with the time pressure. But also, some people don't have the money or the access to technology or the Internet, so therefore paper might be a lot easier. -P011
	**Available Resources**	**Time**	It is a difficult one with PROMs because they are going for personal questions. It needs to be an environment where you can ask for the help if you want it, but if you want that privacy, the helper leaves the room or goes and gets a cup of coffee or whatever. - P003

**Individuals**	**Implementation Team Members**	**Peer Support**	Just letting them [patients] know that if any kind of gender service has a peer support network, letting them know that that's available if anything on the PROM is more distressing to them, there's people that they can talk to. - P011
	**Other Implementation Support**	**Organizations**	I'm autistic and have ADHD, and I personally sometimes struggle to fill in forms. Pointing people to some organizations that could be of help might be useful. So, Citizens Advice is the most neutral one, but there could also be some like LGBT specific ones maybe depending on the area. – P014
**Implementation Process**	**Assessing Needs**	**Innovation Recipients**	You have to be able to take the PROM and say, look, you know, I'm not asking the computer to fill this in - I'm asking a person to fill this in. So what does that individual person need, not what does the PROM need because the PROM shouldn't be the thing that we're worried about, it should be the person that's filling it in. - P009
			It is about person-centred care. I mean, I understand the need to have robust clinical tools that can be applied across and that can actually show differences between people pre- and post-interventions, but it's about person-centred care, it's always gonna boil down to that. Essentially that people will have different needs, different accessibility needs that kind of thing and they will need to be asked what they are. - P008
			So I wouldn't know how best to implement PROMs, but I feel like it does need to be tread very delicately of how this PROM is being approached because you're either going to get data that's a gamified answer or you're just going to get data that's somehow being misled, or just imitating and offending the people answering the questions, I think that's where drawing that fine line is gonna be the biggest challenge. - P008
			Say for example, I get appointments every four months and even if it was a PROM at the four-month mark, for me personally, my answers are not going to change much at all - longer still would be more accurate, depending on how frequently you meet with your care team and how quickly you personally are progressing. Because I think obviously, each person has a need for different time periods where you fill PROMs out. - P006
	**Engaging**	**Innovation Deliverers**	I think there is a role for having a clinician involved in communicating about the PROM, partly for the benefit of the patient to have things explained. - P001
	**Tailoring Strategies**	**Timing**	Immediately after being like you just had your appointment, 'Here's a PROM' wouldn't work because for quite a lot of people, the sessions that they go to are quite distressing and emotional, and that's not something you want to immediately put yourself into doing is filling in a PROM. - P011

Appendix 4. Additional Healthcare Professional Supportive Quotes on Gender-Affirming Care PROM Implementation

CFIR Domain	CFIR Construct	Sub-Construct	Supporting Quote(s)
**Innovation**	**Innovation Complexity**	**PROM**	The complexity of some PROMs would make it almost near impossible to doing a consultation - there's no way you could get through and meaningfully talk about it. But like the ones that I've seen in the past, maybe three or four or five questions, that could provide - like if it was thought about very clearly - could be helpful. – S008
			It does add to the complexity and the burden of the consultation for the patient and for us [clinicians] as well because it's another thing to talk about and it's already quite a complicated consultation to start with, for many people. And I think that's OK, if there's some really clear usefulness of it. – S010
			Sometimes PROMs are just so long, and people will just quit halfway through it. – S004
			And the other thing is getting that balance of how often to get them [patients] to do it, to make sure we're getting the data enough to warrant its [PROM] usefulness but not to be overburdened by it. So, thinking about the intervals between collection sets as well. How useful it is, how soon, how long? – S003
		**Score**	I will say that a PROM needs to be easy to add up and calculate. I've done some of the psychological measures before, where, like, oh, my God, you need a PhD in maths to try and figure out what the score is. I would like a PROM to be very straightforward and easy to score. I also used one and you had to go online and score it. It was honestly a nightmare. – S008
			I think the idea of having an autonomous service for scoring is actually a good idea, because it takes away all the administrative hassle of having to do it. – S002
			I can understand graphs. Yeah, but I could not if I had to do calculations all of that. – S009
			If scoring is done as something that we could click on and see the whole of the graph and how it's working out, that'd be fantastic. If it was another thing that we had to hunt through billions of documents to find and understand the process before we started the work, that would just be a burden.– S010
	**Innovation Design**	**Accessibility**	Making PROMs accessible to all groups, including people with intellectual disabilities or lower literacy skills, or making easy read versions is important. – S005
			And perhaps if you could have spoken voice if need be. – S009
	**Innovation Cost**		When you're on about the untameable costs, it's if something goes wrong. It's the cost about reputation as well. You know it then puts us into a negative way and people then aren't trusting of us and therefore the more they might be more reluctant to access our service. So we've got to look at it, not just the corporate reputation of [trust], but also with [clinic].-S003

			It's all of these things that are unseen and people don't necessarily think about the doctor's time, the clinical time it costs for the person to sit and explain it to them, the cost it takes to send out any surveys. And the cost of the paper, the cost of the letter, the cost of postage returned, the time too, and if it is going to be taken from one system to another, if it has to be done manually, then that's another person's time. – S002
			How is this going to be interpreted by, say people in the NHS, organizations, or higher ups it. It will be a big ethical consideration in terms of like how do we try and harness to make sure that this does not cause any damage.-S004
**Outer Setting**	**Local Conditions**	**Political Environment**	There is always paranoia with what you are going to do with this really personal information of mine. I see that's increased over recent times and I think it's because of the stuff that happens within politics and the media at the moment. People, you know, are much more on hyper alert for that. – S005
	**Critical Incidents**	**Responses**	Or if a PROM is sent out beforehand, somebody completes it, sends it back to admin and then it looks very challenging if lots of things are scored highly on - then it has to be from a risk perspective, and a duty of care, would then be having to end up dealing with it before the patients actually arrive for the consultation. – S008
			The problem with [open text box for comments at the end of PROM] is if the patient writes, ‘I'm going to kill myself’. You know what? We're gonna pick that up and what are we gonna do with that? – S010
**Inner Setting**	**Communications**	**PROM Information**	I think basically every single possible way that you can communicate on what PROMs are and what they are used for, you need to communicate it because people learn and take in information in very different ways and so I think you need to reinforce that message in every possible way that you can at every opportunity that you get. So, if you're sending out written information, it needs to be a paragraph. If you need more information, click on this link and you can watch a video. If you'd like to talk to someone about this, then please call. If you have any face-to-face meetings then it needs to be the first thing that you say is just to reiterate, make sure that everybody is aware that this is what this is, what it's for. This is why we're doing it, and if you have any issues with that then please you know. Communicate with us about it and let us help you. So every possible way that you can.-S002
			Making it clear how PROMs are going to benefit and help and why we're collecting this data and making it clear like we're not just collecting it for fun, what we're trying to achieve from it, I think will really help. -S004
			I'm wondering about disclaimers, so sort of saying this isn't this isn't something that has any political alignment or political agenda or anything. – S005
			Having it really clear about what the purpose of PROMs are or that you know if it is about evaluating the service that any you know, any acute problems they've got are not to be written on this form. – S008

			So definitely if we're going to do it, you really need to help us to understand why we're doing it. Whether it's for the service to benefit or the patient or what it is, but that stage of change management is not complete in my view. – S010
	**Access to Knowledge and Information**	**Benefit to Service Provision**	I would hope it comes back to that initial conversation we were having, and I think it would be true of the clinicians as much as it would be of the clients at the clinic that it is important that they can see the value in it [PROMs] and they understand the purpose of it. -S002
			I get why outcomes are important and. And I think I don't think it would take much explanation to get the rest of the team to get them on board.- S005
			So my initial thought is like this kind of data, if we were able to create something, it would really be invaluable. Like currently there's not really this kind of data collected and like it would really show hopefully that we're helping and how we're helping and doing things. So see if waiting longer, does that actually decrease life satisfaction and actually being able to quantitatively prove a lot of these things, well not prove, to show a lot of these things I think would be very valuable, not just for research, but for clinically, so that we can go to NHS England, for example, and show like the data. - S004
	**Culture**	**Recipient-Centeredness**	You have to be really careful not to out a patient. So, if you sent it to an old address and they and they opened it, then they might say, 'Oh my goodness, I didn't realise they went to a gender clinic'. – S010
	**Structural Characteristics**	**Physical Infrastructure and Information Technology Infrastructure**	And I've been supportive of the electronic system depending on how much hybridity there is and how much support you might need from an app. Because if you've got a purely electronic system, you can automate those like alerts and stuff. Or you can automate it being sent out. – S004
			Having electronic and hard copy available is important, because not everyone has an e-mail or wants to use the computer or can afford Internet. – S007
	**Available Resources**	**Time**	Unfortunately, I think a lot of the time, the system doesn't support it, and it's not 5 minutes, it's 10 minutes. It's 15 minutes. And then if the system is down for any length of time, that means going back after your next appointment trying to remember what was said at the first appointment, so you review the PROM and that means your day is extended by half an hour, an hour, whatever. If you don't do it that same day, then it gets taken over to the next day. If you only work part time, it gets taken over to the next week and then suddenly you get this snowball where you know I can see that that would be a concern. – S002
			It is looking at appropriate use of time and it's going to take time away from that face-to-face contact with clients. – S004
**Individuals**	**High-level Leaders**	**Senior Management**	You need to get to senior management buy into this. So, it's not just seen as something that our that little Gender Clinic's gone off on a tangent again and done something a bit

			different, it needs to be something that's seen as being part of a strategy that's supported by the entire trust, and legitimate within the entire trust as something that is seen as being valid, because otherwise you know they won't be buying into it. – S002
	**Implementation Facilitators**		But I think there does need to be human behind PROMs, so it doesn't feel like we're just cold robots asking for your data. This [sending out PROMs] kind of aligns with the assistant psychologist job. -S004
			Or I don't know if admin could support with this. I think if we are going to send out paper copies, there will need to be admin support to send those out at the appropriate points and depending on how often we decide for these to be done-S003
			And even if it was only like a 15-minute time slot, having a dedicated member of staff who doesn't have any other role, could be a trained up. We're hopefully getting a like a medical assistant, but a medical secretary kind of person. But someone like that who has enough knowledge and skills to know what it's all about can deliver that, but has time and space. Where they can say you know there's X amount of clinicians doing appointments that day, and so there'll be 15 people needing this today. Here's your room. Here's your - it's all and it could be, you know, I'm just thinking who else could do this kind of role to take the burden off, but also ensure it gets done properly and within giving it the importance that it's due. Rather than trying to probe it in or squeeze it in or do it as an afterthought in, especially if some appointments are difficult or challenging or Breaking Bad news, it's oh, by the way, can you do this now? Isn't going to be particularly useful for anyone.-S001
			But then if someone is coming for a clinical appointment and there's nobody to support them to fill in the form, then either it falls on the clinician, which is not, I don't think is a good use of clinical time, or there needs to be an additional person who's available and flexible at that moment of the appointment when that finishes to instantly sit down with that person and help them and support them. -S002
**Implementation Process**	**Assessing Needs**	**Innovation Recipients**	It would be worth having when the first initial contact made, have a question say, 'Do you have any specific needs? Do you need the PROM adapted into specific formats? Please let us know what you would like and then we can change the text, send different text size, different colour.' We can make sure it's really readable, so asking the person first. -S003
	**Engaging**	**Innovation Recipients**	Having patients feel as though they're part of it. They'll benefit from it. And if they are gonna gain from this, then they will feel more engaged to do it and take part in the PROM, where the thing it's just us doing it for the sake of it, for the number crunch and it will be very hard for them. – S003
			And well, but the other thing I was thinking about was whether some people might feel reluctant to engage if they didn't really understand why it was being done or kind of what the purpose of it was or if it might be used in some negative way which might be

			like just - that might not be realistic. I mean, it might not be a grounded fear, but I guess there would need to be some explanation about it for people. – S006
			Because I think if I were to have a one-to-one conversation with a patient about it and explain why it's happening, I think there would be more buy in to it, but realistically, are we gonna be able to do that? – S005
	**Reflecting & Evaluating**		I think it would be good to have a regular focus group with patients, like, every six months or something just to see what they think. Because I think with something like this, it's not ever gonna be just one solution or one like a one-time thing. It needs to be sort of a continuous evolution. - S004
			Yeah, even if PROM implementation is, you know, maybe going to be evolving, but this is the start of the process or something like that. – S006
